# Cartilage Micrografts as a Novel Non-Invasive and Non-Arthroscopic Autograft Procedure for Knee Chondropathy: Three-Year Follow-Up Study

**DOI:** 10.3390/jcm10020322

**Published:** 2021-01-17

**Authors:** Marco Marcarelli, Marcello Zappia, Lorenzo Rissolio, Chiara Baroni, Carlo Astarita, Letizia Trovato, Antonio Graziano

**Affiliations:** 1 Unit of Orthopedics and Traumatology of Chieri and Moncalieri, Santa Croce Hospital, 10024 Turin, Italy; dottmarcarelli@gmail.com (M.M.); rissolio.lorenzo@aslto5.piemonte.it (L.R.); baroni.chiara@aslto5.piemonte.it (C.B.); 2 Department of Medicine and Health Sciences, University of Molise, 86100 Campobasso, Italy; marcello.zappia@unimol.it; 3 Human Brain Wave, Corso Galileo Ferraris, 63, 10128 Turin, Italy; lab@hbwsrl.com; 4 Sbarro Institute for Cancer Research and Molecular Medicine, Department of Biology, College of Science and Technology, Temple University, Philadelphia, PA 19126, USA; info@hbwsrl.com

**Keywords:** cartilage micrografts, medical device, regenerative medicine

## Abstract

(1) Background: Focal chondral defects of the knee can significantly impair patient quality of life. Although different options are available, they are still not conclusive and have several limitations. The aim of this study was to evaluate the role of autologous cartilage micrografts in the treatment of knee chondropathy. (2) Methods: Eight patients affected by knee chondropathy were evaluated before and after 6 months and 3 years following autologous cartilage micrografts by magnetic resonance imaging (MRI) for cartilage measurement and clinical assessment. (3) Results: All patients recovered daily activities, reporting pain reduction without the need for analgesic therapy; Oxford Knee Score (OKS) was 28.4 ± 6 and 40.8 ± 6.2 and visual analogue scale (VAS) was 5.5 ± 1.6 and 1.8 ± 0.7 before and after 6 months following treatment, respectively. Both scores remained stable after 3 years. Lastly, a significant improvement of the cartilage thickness was observed using MRI after 3 years. (4) Conclusions: Autologous cartilage micrografts can promote the formation of new cartilage, and could be a valid approach for the treatment of knee chondropathy.

## 1. Introduction

Articular cartilage defects remain a challenging clinical issue for orthopedic surgeons and, if left untreated, can progress to degenerative osteoarthritis, knee pain, and ultimately loss of function [1]. The limited self-healing ability of articular cartilage is due to its avascular nature and slow extracellular matrix turnover, along with limited capacity of resident chondrocytes to migrate to damaged areas [2].

Joint preservation is the preferred treatment strategy for knee chondropathologies in young and active patients to improve pain, restore activity, and delay arthroplasty. To date, there have been several approaches that aim the repair cartilage defects, even if the natural course of such lesions is still not well known. Among these, bone marrow stimulation techniques, cartilage or chondrogenic tissue-based repair, and pharmacological approaches have been widely discussed in the literature [3]. Osteochondral autologous transplantation (OAT) is used for small and medium focal articular cartilage defects [4], while autologous chondrocyte implantation (ACI) can be considered for the treatment of early osteoarthritis affecting younger patients [5]. Different generations of ACI and matrix-induced autologous chondrocyte implantation (MACI) techniques have been studied both in long-term follow-ups and in several randomized trials, and these techniques lead to satisfactory results [6]. Although the above-mentioned therapeutics represent an option today, all come with limitations: (i) the need for cell expansion, (ii) high cost, (iii) invasiveness, and (iv) formation of fibrocartilage often occurring by de-differentiation of chondrocytes during cell expansion [7,8]. Pharmacological treatments for knee or hip osteoarthritis include non-selective non-steroidal anti-inflammatory drugs, as well as intra-articular injections of corticosteroids, focusing on pain and inflammation reduction without addressing the underlying causes [9,10].

The aim of this study was to evaluate the clinical effects of autologous cartilage micrografts, delivered with a non-arthroscopic procedure—injection in the articular chamber—on eight patients affected by knee cartilage degeneration and evaluated by magnetic resonance imaging (MRI) and clinical assessment before and after 3 years following treatment.

For this specific aim, we used the Rigeneracons medical device, an innovative technology that allowed us to mechanically disaggregate autologous cartilage, collected from the patient, to obtain a micrografts suspension ready to use without the need for cell expansion in vitro and able to regenerate damaged tissues. To date, the Rigenera^®^ micrografting technology has demonstrated its efficacy in different clinical settings. It is widely used with dermal tissue samples for the treatment of complex wounds such as dehiscences, chronic ulcers, and burns [11,12,13,14,15,16], for dermatological applications such as hypertrophic scars [17], and for the management of androgenetic alopecia [18,19], with cartilage samples for the management of chondropathy [20,21], with bone samples for the treatment of the osteonecrosis of the femoral head [22].

## 2. Experimental Section

### 2.1. Research Design

The study protocol was in accordance with the Declaration of Helsinki of 1975 and European regulations. All patients provided written informed consent before participating in the study. Eight patients were enrolled. Their ages were between 43 and 69 years old affected by knee degenerative chondral lesions, pain for more than 3 months following conservative treatment, and no bone axial defect. Patient diagnosis was accomplished with the Outerbridge classification [23]. Patient inclusion criteria were knee chondropathy grade I to III (pre-arthrosis). The exclusion criteria were chondropathy grade IV, several osteophytosis, traumatic or degenerative meniscal lesions, and systemic disease such as rheumatoid arthritis and psoriatic arthritis.

All patients reported in Table 1 were treated with a single intra-articular injection of autologous micrografts obtained with the Rigenera^®^ micrografting technology. The Oxford Knee Score (OKS) and the visual analogue scale (VAS) were used before and after 6 months following micrografts treatment to perform a global assessment of patient satisfaction, pain decrease, and functional status [24].

MRI of the knee was performed in patients before and after 3 years following single micrografts application to evaluate the status of cartilage degeneration/regeneration.

To measure the thickness of cartilage, DP, weighted or intermediate-weighted, was used with fat saturation sequences (serial checks always made on the same sequence). The slice thickness was 3 or 4 mm. The measurement of the cartilaginous thickness of the central portion of the condyles was done on the coronal plane (in the middle of the condyle on the sagittal plane).

The measurement of the posterior portion of the condyles was made on the sagittal plane at 1cm from the end of the cartilage. The point at which cartilage is thickest was chosen and measured. Moreover, if bone edema or focal lesions of cartilage were detected, it would be reported.

### 2.2. Technique

**Figure 1:**
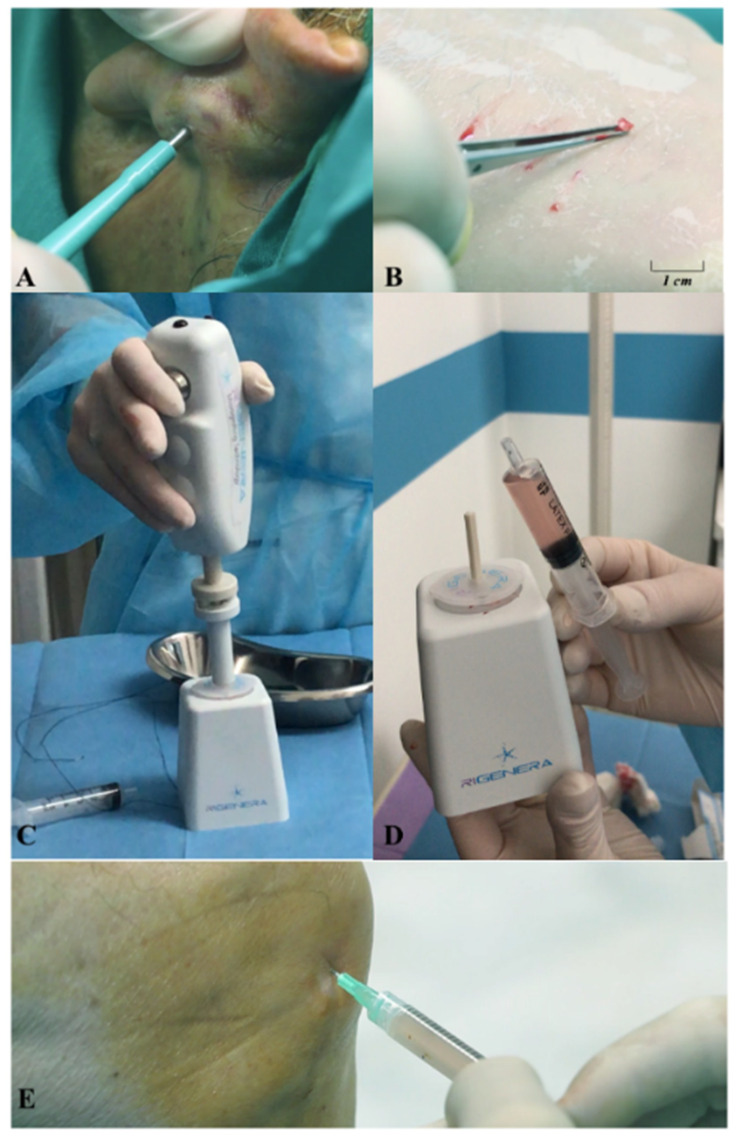
Use of the Rigeneracons medical device to generate autologous cartilage micrografts: (**A**) The autologous cartilage sample is collected with a punch; (**B**) Size of the autologous cartilage micrografts after collection; (**C**) The tissue, along with sterile saline solution, is inserted in the Rigeneracons device, which is activated by its motor; (**D**) After the cartilage fragmentation, the micrografts suspension is directly collected; (**E**) The cartilage micrografts suspension is directly injected into the knee of the patient with a syringe.

A small piece of auricular cartilage was mechanically disaggregated immediately by the Rigeneracons medical device (Human Brain Wave, Turin, Italy), which can generate a micrografts suspension with a cut-off of 80 microns, making it possible to deliver such graft suspension with a simple syringe into the synovial capsule. More specifically, an autologous auricular cartilage sample (one for each knee) is easily collected by a 3 mm punch biopsy (Figure 1A,B); the autologous cartilage is then placed into the medical device, adding 4 mL of sterile saline solution. Afterwards, the Rigeneracons is activated with a specific motor for 4 min, allowing the rotation of the inner blades responsible for the micro-fragmentation of the cartilage autologous sample (Figure 1C); such cartilage micro-fragments (or cartilage micrografts) are now in suspension with sterile saline and are easily collected form the device with a syringe (Figure 1D). Lastly, the cartilage micrografts suspension is injected with a syringe by anterolateral approach in the knee of the patient (Figure 1E).

### 2.3. Statistical Analysis

Statistical analysis was performed by using GraphPad Prism software. Data were expressed as the mean ± standard deviation (SD). The difference between parameters was examined using an independent-samples or paired-samples t-test. A *p* ≤ 0.05 was considered statistically significant.

## 3. Results

In all cases, the procedure was successful, and none of the patients experienced infections or severe complications after the procedure. No aesthetic defects were observed at the donor site, and none complained about the results. All patients reported a pain resolution and a good recovery of daily activities (Table 2).

### 3.1. Oxford Knee Score (OKS) and the Visual Analogue Scale (VAS)

From a clinical point of view, all patients recovered their daily activities without the need for analgesic therapy as indicated by OKS and VAS (Table 3). The mean preoperative and postoperative OKS was 28.4 ± 6 (range, 19–36) and 40.8 ± 6.2 (range, 30–47), respectively.

The VAS was 5.5 ± 1.6 and 1.8 ± 0.7 before and after 6 months, respectively. Both OKS and VAS values remained stable also after 3 years.

### 3.2. Magnetic Resonance (MRI) and Cartilage Mesuraments

**Figure 2:**
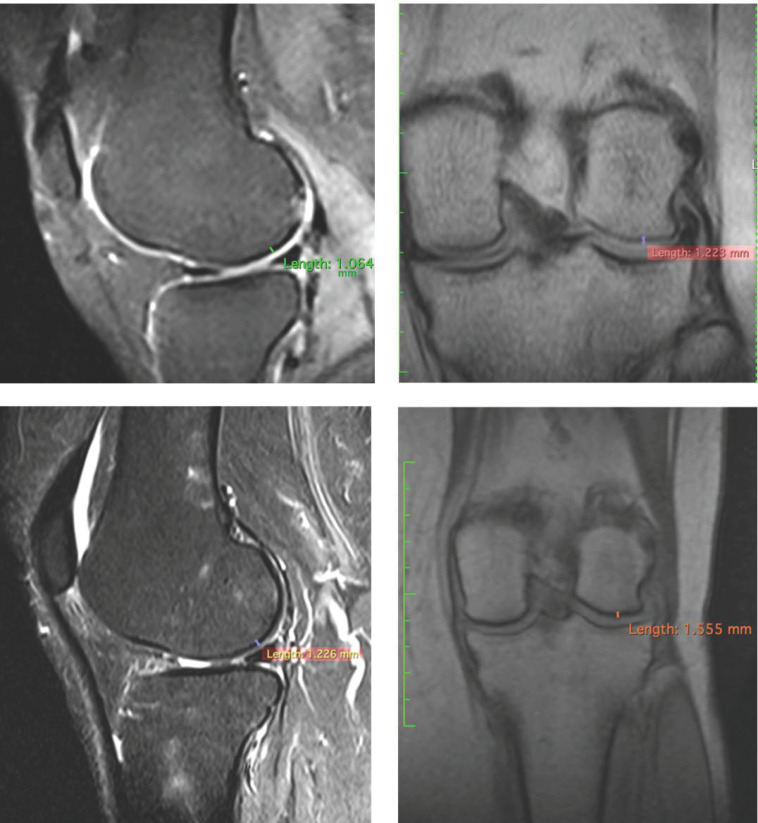
Representative images of MRI of patient no. 1 at 3 years. Images of femoral lateral condyles and measurement of thickness cartilage. We state that different MRI machines and different sequences were used for each patient.

**Figure 3:**
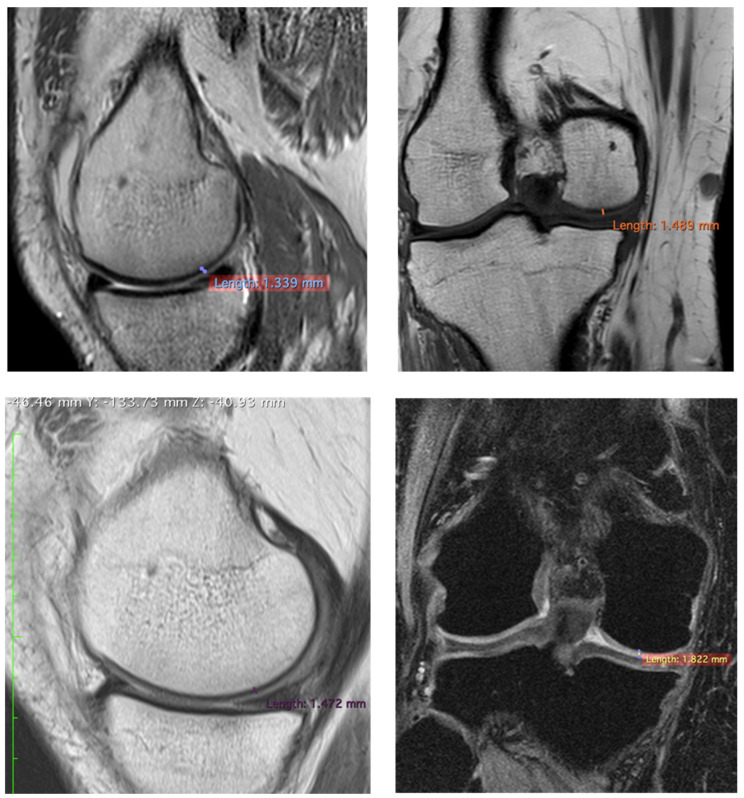
Representative images of MRI of patient no 6 at 3 years. Images of femoral lateral condyles and measurement of thickness cartilage. We state that different MRI machines and different sequences were used for each patient.

**Figure 4:**
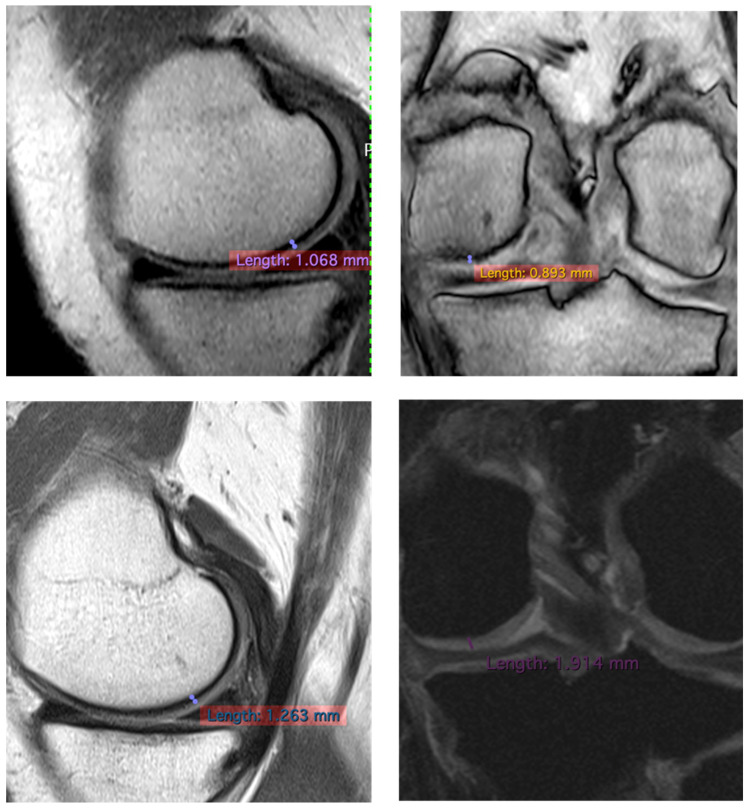
Representative images of MRI of patient no 2 at 3 years. Images of femoral lateral condyles and measurement of thickness cartilage. We state that different MRI machines and different sequences were used for each patient.

To confirm the positive clinical outcome, as reported by the OKS and VAS score, we evaluated the cartilaginous thickness of the patients undergoing the micrografts application by MRI, which was performed at baseline and after 3 years. Interestingly, we observed an increase in thickness of the femoral lateral condyles on the sagittal and coronal plane (Figure 2, Figure 3 and Figure 4). The mean measurements of femoral condyle on the sagittal plane were 1.2 mm (±0.2) at baseline and 1.3 mm (±0.1) after 3 years, showing an increase of 10% (*p* = 0.006), as reported in Table 4. For the coronal plane, the mean length of femoral condyle was 1.2 mm (±0.3) at baseline and 1.8 mm (±0.2) after 3 years showing an increase of 40% (*p* = 0.025), as shown in Table 5.

## 4. Discussion

Cartilage lesions have a wide variety of causes and may result in the persistence of important alterations in the skeletal plane. Some lesions, after the removal of the etiological agent, have the possibility to be repaired by natural biological processes. This outcome is only achievable in a few cases because of limited self-healing ability of the cartilage. On the other hand, this process could take years to complete a repair, which becomes a complicating factor for the resolution of the entire process.

Current intervention can be grouped into biologic-based therapeutics as Hyaluronic Acid (HI) or Platelet-Rich Plasma (PRP), surgical intervention, and cell-based approaches [25].

In this study, were reported preliminary data and exploited the use of cartilage autologous micrografts in the treatment of knee chondropathy, showing clinical improvement in terms of quality of life of the patients (Table 3) and, most importantly, patients who have undergone MRI analysis show an increase in thickness of cartilage (Figure 2, Figure 3 and Figure 4). MRI analysis was selected as the standard to evaluate the cartilaginous thickness, compared to X-ray evaluation, which would allow the discrimination of only the intra-articular space and therefore an indirect information on the cartilage status as described in the literature [26]. Even though the analysis was performed with different MRI machines, this is not an issue, since both had a 1.5 Tesla (T) scanner and the parameter analyzed was only dimensional.

The analyses here reported were evaluated by an expert radiologist, standardizing the MRI plane to evaluate the cartilaginous thickness.

The novelty of this approach is related to the technology applied, the Rigeneracons, which allowed mechanical disaggregation of the small sample of autologous cartilage, generating a cartilaginous tissue suspension ready to be grafted onto the patient with a simple syringe (Figure 1).

It should be noted that the autologous micrografts used in this study were immediately available, since the procedure lasted 10 min, without the need for culture/differentiation in vitro. Furthermore, previous studies have demonstrated that autologous micrografts generated from auricular cartilage were positive for cartilage progenitor cell markers such as CD44, CD90, and CD117; moreover, the micrograft-derived RNA was quantified and positive for tissue cartilage markers such as Sox-9 and COL2A1 [27]. In vitro studies also reported that the Rigenera Cartilage-Derived Micrografts can influence chondrocyte growth by (i) increasing glycosaminoglycans (GAGs) deposition, (ii) enhancing the production of Collagen II and (iii) the presence of IGF-1 and TGFβ within the micrografts suspension [20]. The clinical application of those micrografts was also reported in another independent study where all patients reported a remarkable pain decrease during the few days following the procedure, suggesting an immunomodulatory effect mediated by micrografts even if other studies are required to demonstrate this [21].

The use of micrografts, with respect the use of cell-based techniques such as ACI or MACI, overcomes the limitations related to these methods. In fact, the ACI or MACI techniques are based on the in vitro cell expansion of chondrocytes, which can lead to the formation of fibrocartilage due to the de-differentiation of chondrocytes during cell expansion [28]. Additionally, the proliferative rate of chondrocytes decreases with the age of the donor, limiting the applicability of ACI or MACI techniques [29].

Another promising approach in cartilage repair is represented by mesenchymal stem cell (MSC) application due their high index of differentiation and facility in collection from different sources including bone marrow, adipose tissue, synovial membrane, and others [30,31]. MSCs also have an extensive potential to differentiate into chondrocytes, exerting anti-inflammatory and immunomodulating properties, offering a promising tool in cartilage repair [32]. However, the transplantation of MSCs often gives rise to a mixture of hypertrophic, cartilaginous, and fibrous tissues, which, in the long run, leads to a loss of repair tissue [33].

OAT (mosaicplasty) is a tissue-based option suitable for patients with lesions of a substantial size (>3 ${\mathrm{cm}}^{2}$). In this procedure, which can be performed arthroscopically, grafts are collected from the periphery of the non-weight-bearing trochlea, then a drill guide is inserted perpendicular to the joint surface in the defect to allow graft sockets to be reamed. The OAT has shown encouraging results for up to 10 years after surgery; however, the additional effect over traditional microfracture treatment is reduced over time [34].

Lastly, compared to non-surgical intervention (PRP or Hyaluronic Acid) the autologous cartilage micrografts retain the same simplistic approach in terms of delivery (injections in the articular space); however, being autologous living tissue, the cartilage grafts represent the conjunction with surgical-based intervention (OAT), up to now rarely used because of its extreme invasiveness.

## 5. Conclusions

The use of the Rigenera micron-sized (80 micron) micrografts delivered with a simple syringe is increasingly expanding in different clinical settings with the possibility for use in different tissues. In the context of knee chondropathy (grades I to III), a single application of autologous cartilage micrografts improved the quality of life of patients, since no analgesic therapy was needed and, most importantly, it promoted the formation of new cartilage. This novel autograft procedure represents an innovative and accessible approach for the management of chondropathy; however, considering the small sample size, more studies are necessary to validate such clinical application. Lastly, this approach could be expanded to different joints over the human body, as our group have successfully treated the hip joint and ankle joint by using the same criteria here reported. However, for other cartilaginous pathologies (i.e., arthritis) more in vitro analysis should be performed to monitor if and how the micrografts have a positive outcome, possibly reducing the chronic inflammation in this different clinical context. The major limitations of this study are: (i) the lack of standardization for MRI; and (ii) the absence of a control group. However, it should be defined what would a suitable control group, if non-surgical (palliative) approaches such as PRP and HI or tissue-based approaches, surgical approach as OAT are acceptable. A suitable comparison would be with the surgical/tissue-based approaches such as OAT; however, such procedures are rarely preferred, as they are extremely invasive.

## Figures and Tables

**Table 1:** Diagnosis and type of treatment of the patients included in the study.

Patients	Sex, Age (y)	Diagnosis	Treatment
1	F, 66	Chondropathy grade III	Micrografts collection from auricular cartilage and application on left knee
2	F, 53	Chondropathy grade III	Micrografts collection from auricular cartilage and application on left knee
3	F, 60	Chondropathy grade III	Micrografts collection from auricular cartilage and application on left knee
4	M, 54	Chondropathy grade III	Micrografts collection from auricular cartilage and application on both right and left knee
5	F, 55	Chondropathy grade II	Micrografts collection from auricular cartilage and application on right knee
6	M, 69	Pre-arthrosis	Micrografts collection from auricular cartilage and application on both right and left knee
7	M, 43	chondropathy grade III	Micrografts collection from auricular cartilage and application on right knee
8	M, 50	chondropathy grade III	Micrografts collection from auricular cartilage and application on right knee

**Table 2:** Clinical outcomes of patients included in the study.

Patients	Clinical Outcomes
1	Complete pain resolution, recovery of daily activity
2	Complete pain resolution, general improvement about 6 months
3	Complete pain resolution, recovery of daily activity
4	Pain resolution, general improvement, and recovery of daily activity after 1 month following treatment
5	Complete pain resolution, general improvement, and recovery of physical and daily activity after 1 month following treatment
6	Complete pain resolution, general improvement, and recovery of physical and daily activity after 6 months following treatment
7	Complete pain resolution
8	Complete pain resolution, general improvement, and recovery of physical activity

**Table 3:** Clinical outcomes of patients included in the study.

Patients	OKS Pre	OKS Post	VAS Pre	VAS Post
1	19	30	5	3
2	22	33	6	2
3	25	40	7	2
4	28	41	8	2
5	31	45	4	1
6	32	44	3	1
7	34	46	5	2
8	36	47	6	1

**Table 4:** MRI results of cartilaginous thickness of femoral lateral condyles on the sagittal plane.

Patients	Pre/Femoral Lateral Condyles	3 Years/Femoral Lateral Condyles	Difference
1	1.064 mm	1.226 mm	+15.22%
2	1.339 mm	1.472 mm	+9.32%
3	1.068 mm	1.263 mm	−14.20%

**Table 5:** MRI results of cartilaginous thickness of the femoral lateral condyles on the coronal plane.

Patients	Pre/Femoral Lateral Condyles	3 Years/Femoral Lateral Condyles	Difference
1	1.223 mm	1.555 mm	+27.14%
2	1.489 mm	1.882 mm	+26.39%
3	0.893 mm	1.914 mm	+114.33%

## Data Availability

Data sharing not applicable.
